# Supplementary material for: HIVprotI: an integrated web based platform for prediction and design of HIV proteins inhibitors
Source: J Cheminform. 2018 Mar 9;10:12. doi: 10.1186/s13321-018-0266-y (PMC5845081; doi:10.1186/s13321-018-0266-y)
Supplement: Supplementary file 2 — Additional file 2. Source code of HIVProtI web server. [file 13321_2018_266_MOESM2_ESM.zip › HIVprotI_Source-code/hivprotI/weka-3-6-6/doc/weka/gui/SelectedTagEditor.html]

SelectedTagEditor


---


|  |  |  |
| --- | --- | --- |
| **Overview** \| **Package** \| **Class** \| **Tree** \| **Deprecated** \| **Index** \| **Help** \| **Weka's home** | |  |
| **PREV CLASS**   **NEXT CLASS** | **FRAMES**    **NO FRAMES**     **All Classes** |  |
| SUMMARY: NESTED \| FIELD \| CONSTR \| METHOD | DETAIL: FIELD \| CONSTR \| METHOD |  |


---


## weka.gui Class SelectedTagEditor

```
java.lang.Object
  java.beans.PropertyEditorSupport
      weka.gui.SelectedTagEditor
```

**All Implemented Interfaces:**: java.beans.PropertyEditor

---

``` public class SelectedTagEditor extends java.beans.PropertyEditorSupport ```

A PropertyEditor that uses tags, where the tags are obtained from a
weka.core.SelectedTag object.

**Version:**
:   $Revision: 7059 $

**Author:**
:   Len Trigg (trigg@cs.waikato.ac.nz)

---

**Constructor Summary**

`SelectedTagEditor()`


| **Method Summary** | |
| --- | --- |
| `java.lang.String` | `getAsText()`             Gets the current value as text. |
| `java.lang.String` | `getJavaInitializationString()`             Returns a description of the property value as java source. |
| `java.lang.String[]` | `getTags()`             Gets the list of tags that can be selected from. |
| `static void` | `main(java.lang.String[] args)`             Tests out the selectedtag editor from the command line. |
| `void` | `setAsText(java.lang.String text)`             Sets the current property value as text. |

**Methods inherited from class java.beans.PropertyEditorSupport**

`addPropertyChangeListener, firePropertyChange, getCustomEditor, getSource, getValue, isPaintable, paintValue, removePropertyChangeListener, setSource, setValue, supportsCustomEditor`

**Methods inherited from class java.lang.Object**

`equals, getClass, hashCode, notify, notifyAll, toString, wait, wait, wait`

**Constructor Detail**

### SelectedTagEditor

```
public SelectedTagEditor()
```


**Method Detail**

### getJavaInitializationString

```
public java.lang.String getJavaInitializationString()
```

:   Returns a description of the property value as java source.

    :   **Specified by:**: `getJavaInitializationString` in interface `java.beans.PropertyEditor` **Overrides:**: `getJavaInitializationString` in class `java.beans.PropertyEditorSupport`
    :   **Returns:**: a value of type 'String'

---


### getAsText

```
public java.lang.String getAsText()
```

:   Gets the current value as text.

    :   **Specified by:**: `getAsText` in interface `java.beans.PropertyEditor` **Overrides:**: `getAsText` in class `java.beans.PropertyEditorSupport`
    :   **Returns:**: a value of type 'String'

---


### setAsText

```
public void setAsText(java.lang.String text)
```

:   Sets the current property value as text.

    :   **Specified by:**: `setAsText` in interface `java.beans.PropertyEditor` **Overrides:**: `setAsText` in class `java.beans.PropertyEditorSupport`
    :   **Parameters:**: `text` - the text of the selected tag. **Throws:**: `java.lang.IllegalArgumentException` - if an error occurs

---


### getTags

```
public java.lang.String[] getTags()
```

:   Gets the list of tags that can be selected from.

    :   **Specified by:**: `getTags` in interface `java.beans.PropertyEditor` **Overrides:**: `getTags` in class `java.beans.PropertyEditorSupport`
    :   **Returns:**: an array of string tags.

---


### main

```
public static void main(java.lang.String[] args)
```

:   Tests out the selectedtag editor from the command line.

    :   **Parameters:**: `args` - ignored


---


|  |  |  |
| --- | --- | --- |
| **Overview** \| **Package** \| **Class** \| **Tree** \| **Deprecated** \| **Index** \| **Help** \| **Weka's home** | |  |
| **PREV CLASS**   **NEXT CLASS** | **FRAMES**    **NO FRAMES**     **All Classes** |  |
| SUMMARY: NESTED \| FIELD \| CONSTR \| METHOD | DETAIL: FIELD \| CONSTR \| METHOD |  |


---
